# Patient satisfaction with telemedicine for prechemotherapy evaluation during the COVID-19 pandemic

**DOI:** 10.2217/fon-2020-0855

**Published:** 2021-02-26

**Authors:** Ajithraj Sathiyaraj, Hannah Lopez, Rakesh Surapaneni

**Affiliations:** ^1^Department of Internal Medicine, Baylor Scott & White Medical Center, Round Rock, TX 78665, USA; ^2^Department of Hematology & Oncology, Baylor Scott & White Medical Center, Round Rock, TX 78665, USA

**Keywords:** COVID-19 pandemic, patient satisfaction, pre-chemotherapy evaluation, telehealth, video visits

## Abstract

**Aims:** This project aims to address the question of whether patients were satisfied with using a video visit for prechemotherapy evaluation during the COVID-19 pandemic. **Methods and materials:** This project used a survey tool with patients undergoing prechemotherapy evaluation that was administered at the time of chemotherapy; 70 surveys were collected. Descriptive statistics of survey questions are presented. **Results:** 73% of patients reported satisfaction with their video visit experience. 65% of patients reported that they prefer in-person visits as their preferred choice for prechemotherapy evaluation. **Conclusion:** Patient satisfaction was favorable, but not consistent with results from prior published studies. Patients also mostly preferred an in-person visit for prechemotherapy evaluation. Further research is needed to determine patient attitudes to telemedicine for different types of consultations.

## Lay abstract

In this study we looked at how satisfied patients were with video visits to consult with their physicians prior to receiving chemotherapy. We collected 70 surveys from June to July 2020 in the clinic’s infusion center. Most patients were satisfied with using video visits, but maybe were not as satisfied with using video visits as has been reported in other studies. Most patients also still preferred an in-person visit to a video visit. Patients may have preferred in-person visits because that is what they were used to. More research is needed to find why satisfaction with video visits can be so varied.

Telehealth has been proposed as a useful tool in medical systems’ response to disasters [1]. Telehealth, which includes asynchronous e-visits and synchronous audio-only or video visits, has been used by over 9 million Medicare beneficiaries between 17 March and 13 June, 2020 [2]. Many systems have increasingly utilized this tool in many scenarios to interact with patients during recent times due to the COVID-19 pandemic [3]. While many of these cases are necessary for acutely ill patients in areas with shortage of physicians, telemedicine in the outpatient setting poses a different kind of scenario. The Centers for Disease Control guidelines for social distancing, face coverings and infection prevention have been implemented in many clinic settings [4,5]. What was once primarily used for increasing access to care in areas with shortage of physicians has become an alternative to the traditional in-person visit, especially in encounters with patients experiencing symptoms that could be due to COVID-19 or those who prefer to remain distanced during the pandemic. This could reduce the risk of contagion for both health personnel and patient [6,7].

The public health crisis has changed how the clinic visit is conducted, from how patients walk into to the clinic to checking out at the desk. Telemedicine in the form of synchronous video visits can further change these interactions by removing the physician from physically encountering a patient in the traditional pathway that includes an in-person evaluation by the physician. In this study we report patient satisfaction with video visits with their oncologist for prechemotherapy evaluation during the COVID-19 pandemic. This vulnerable population may benefit especially from decreased interactions, which could be accomplished with telemedicine. Since patients have had the added option of scheduling a telemedicine appointment from home, problems that could have resulted in a hospital visit can be managed remotely by the patient’s oncologist [7,8].

Prior studies evaluating patient satisfaction with telemedicine in the outpatient setting have shown that over 90% of patients were satisfied with their video visit experience [9,10]. A systematic review looking at patient satisfaction in the rural setting found that over 80% of patients were satisfied with their video visits when responding to a questionnaire [11]. Regarding telemedicine for cancer care, patients report similar levels of satisfaction [12–15]. A retrospective cohort study using Press Ganey scores to assess predictors for patient satisfaction found that video visits were associated with greater patient satisfaction when compared with in-person visits [16]. A review of patient satisfaction studies conducted during the COVID-19 pandemic also found that patients and providers had high levels of satisfaction with telehealth [17]. A recent prospective cohort using a similar platform to the one used in this clinic showed that over 90% of respondents were satisfied; however, the survey response was limited to 21% of patients [15]. This cohort also did not specify for a particular type of visit, as in this study [15].

This study is unique in that the patients studied were still presenting to the infusion center throughout the pandemic to receive their chemotherapy. The infusion center is also where the patients would usually see their providers for prechemotherapy evaluation, prior to the rapid adoption of video visits. While prior studies have shown favorable outcomes, much of their data relates either to looking at satisfaction in a variety of non-specialty settings or to visit types that are not for prechemotherapy evaluation [9,10,14–16]. Barriers to accessing the clinic are commonly cited reasons in other studies [9,10]. Because telemedicine can include a variety of platforms, review articles usually combine audio-only with video visits [14]. We aim to assess patient satisfaction with video visits for a specific visit type, namely prechemotherapy evaluation, and where access to the clinic was not a barrier, so that oncology practices can determine whether transitioning to primarily video visits is feasible.

Potential downsides for the shift to virtual care of patients include the lack of physical examination and difficulties in showing compassion and empathy through such a medium [15,18]. Patients can feel nervous and anxious surrounding the use of new technologies, which can cause difficulty or reluctance to communicate with providers through telemedicine [8]. Patients also may have a preconceived idea of how a clinic visit is conducted; since the drastic shift to utilizing telemedicine during the early pandemic period, they may have not been prepared to accept this alternative [19].

As a response to the pandemic, the Centers for Medicare and Medicaid Services expanded the use of telemedicine services and changed reimbursement rates for such services to encourage their use [20,21]. The cancer center at our institution adopted these changes and provided patients the option of conducting their prechemotherapy evaluation through a video visit platform; interfaces used included Blue Stream^©^ (FL, USA), and Epic MyChart Video Visits (WI, USA). By conducting a video visit for prechemotherapy evaluation on a day prior to the planned chemotherapy, patients would ideally be able to save time presenting to the clinic and prevent unnecessary travel if they could conduct a video visit from home. The efficiency in the infusion center would also improve because the schedule could be adjusted ahead of the planned session. Patients were also given the option of conducting a video visit utilizing a device in the clinic if they had difficulties using their own device or could not conduct the visit from home. By allowing patients to choose from these options, it was thought that interactions could be minimized while still providing standard of care management for the patient’s malignancy.

## Methods and materials

**Figure 1.**
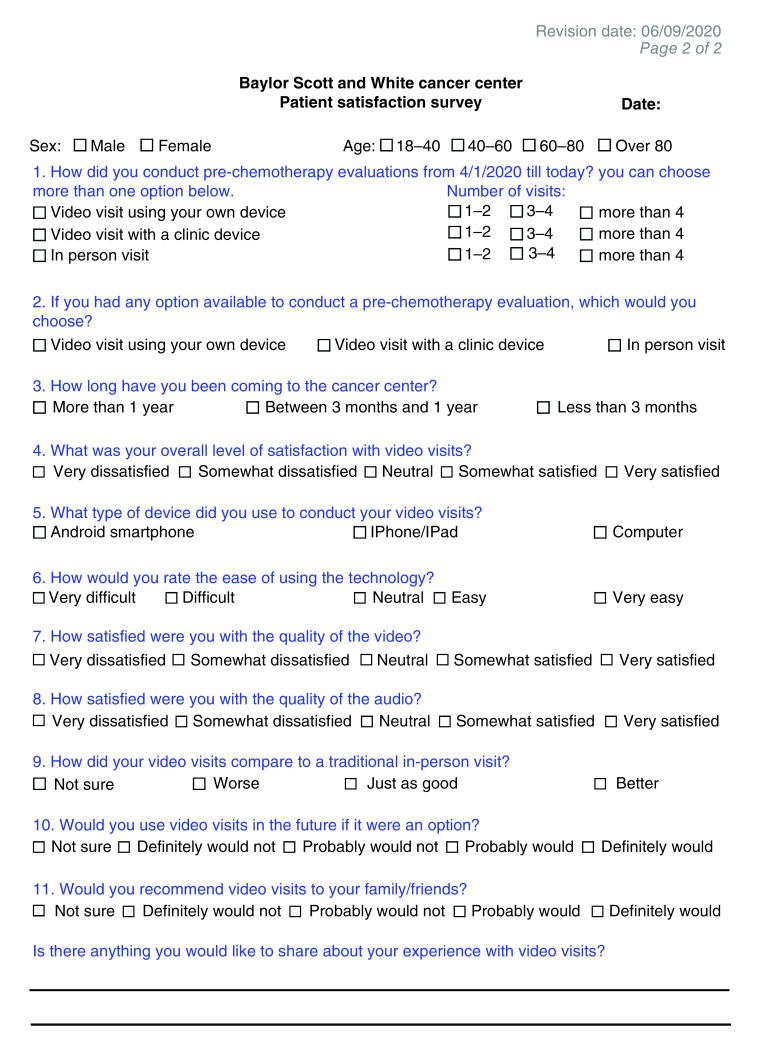
Survey tool used in this study.

This single-center cross-sectional study used a patient survey (Figure 1) created by the study investigators to assess patient satisfaction with video visits for prechemotherapy evaluation. Survey questions from a prior published project were used [9]. Patients were asked to participate in this study by clinic staff in the infusion center or by a study investigator during the time of their chemotherapy. The cancer center has four practicing hematologist/oncologists. Round Rock is a suburb of Austin, Texas and has a population of 118,000, of whom 70.8% report their ethnicity as White/Anglo, with 29% of those representing Hispanic or Latino people [22]. 12.4% of the population does not have health insurance, 6.4% live in poverty, 93% have at least completed high school and 91% of households have an internet subscription [23]. The cancer center also services the surrounding four counties, and some patients come from a more rural setting.

Patients were included in the study if they had conducted a video visit for prechemotherapy evaluation from 1 April 2020 until the time they were asked to participate. Patients were not allowed to participate in the study if they did not have decision-making capacity. Patients who were presenting to the infusion center for other therapies, such as intravenous iron, transfusion of blood products, phlebotomy or injections for osteoporosis or hormone therapy, were not included. Patients were asked to fill an anonymous survey to be included in the study. This study was exempt from formal Institutional Review Board review because it only included interaction involving a survey and the information was recorded in such a manner that the identity of the human subject could not readily be ascertained.

## Statistical analysis

Total unique visits to the infusion center for chemotherapy over the study period were used to calculate a survey response rate. The primary outcome was patient-reported satisfaction with their video visit experience (question 4 of the survey tool). Survey responses were entered into Excel to generate the percentages and figures reported below. The percentage of patients who responded that they were ‘somewhat satisfied’ or ‘extremely satisfied’ is reported. The results of other survey questions are also reported in the corresponding categories (e.g., percentage ‘easy/very easy’, percentage ‘probably/definitely would’).

## Results

Between 1 April 2020 and 14 July 2020, the cancer center had 1405 visits in the infusion center, with 585 unique patients. 241 unique patients presented to the infusion center for chemotherapy and 231 of these patients had at least one video visit that would qualify them for inclusion into the study. The survey was administered between 9 June 2020 and 14 July 2020. The survey completion rate was 30.3% (70/231). Not all 231 patients who qualified for inclusion may have been present at the infusion center during the period in which the survey was conducted. Respondents were predominantly women (67.6%) and between the ages of 40 and 60 (60%). The next major age group represented was between the ages of 60 and 80 (36.9%).

**Figure 2.**
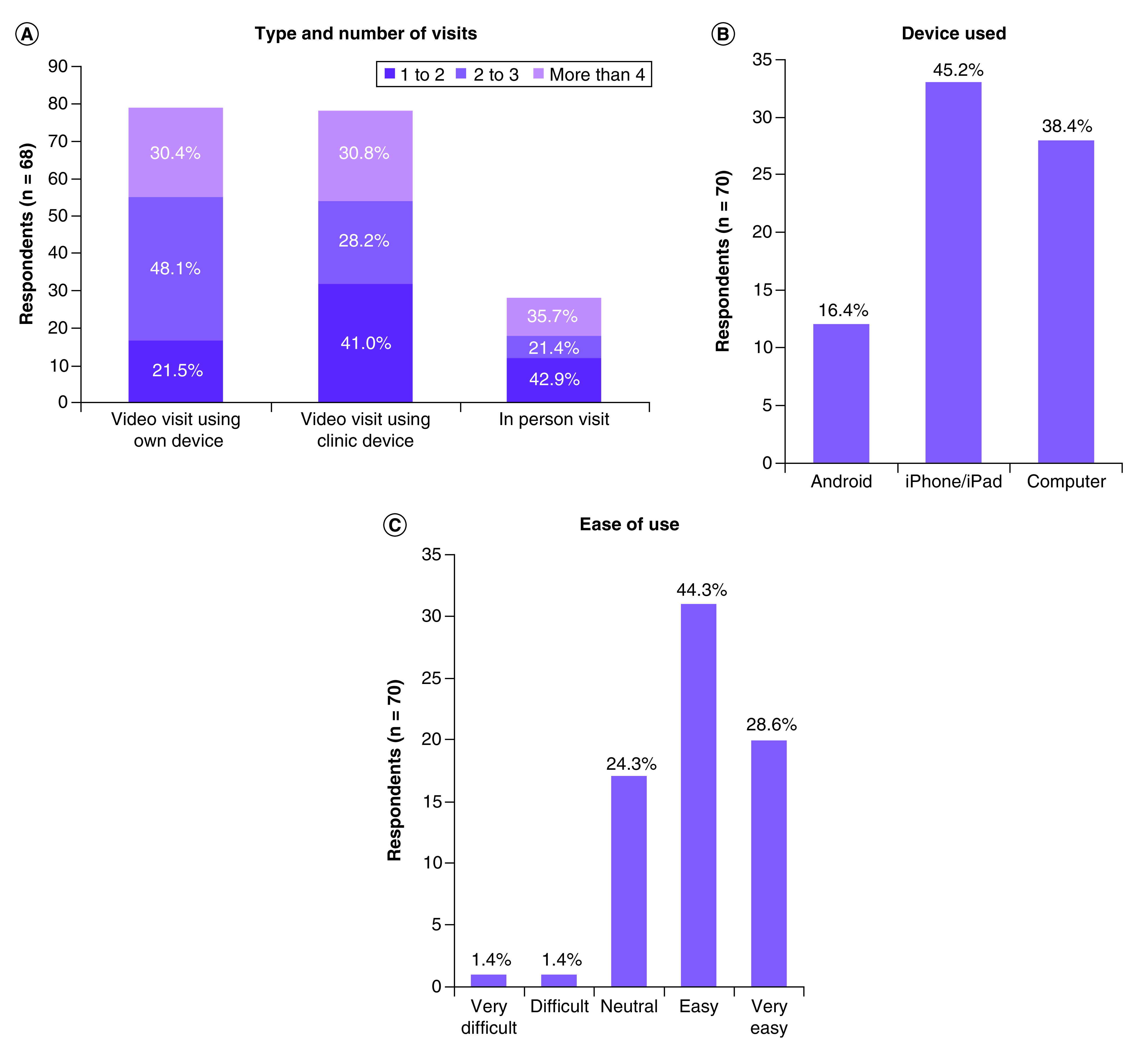
Characteristics of visits and ease of use. **(A)** Type and number of visits patients had during the study period. **(B)** Types of devices used by patients. **(C)** Ease of using telemedicine service.

The method of visit, number of visits, device used and ease of use are shown in Figure 2. 74.6% (n = 67) of patients reported using a video visit with their own device. 44.1% of patients reported conducting a video visit with a device in the clinic. Most patients also had at least one in-person visit with a provider (64.2%) for prechemotherapy evaluation during the period studied. 48.6 % of patients reported coming to the cancer center for between 3 months and 12 months, 41.4% reported coming for more than a year, and the remaining patients reported that they had been coming for less than 3 months. The most common type of device used was an iPhone/iPad (45.2%), followed by computer (38.4%), with the remainder using an Android device. 72.9% of patients also reported finding the technology ‘mostly easy’ or ‘very easy’ to use.

**Figure 3.**
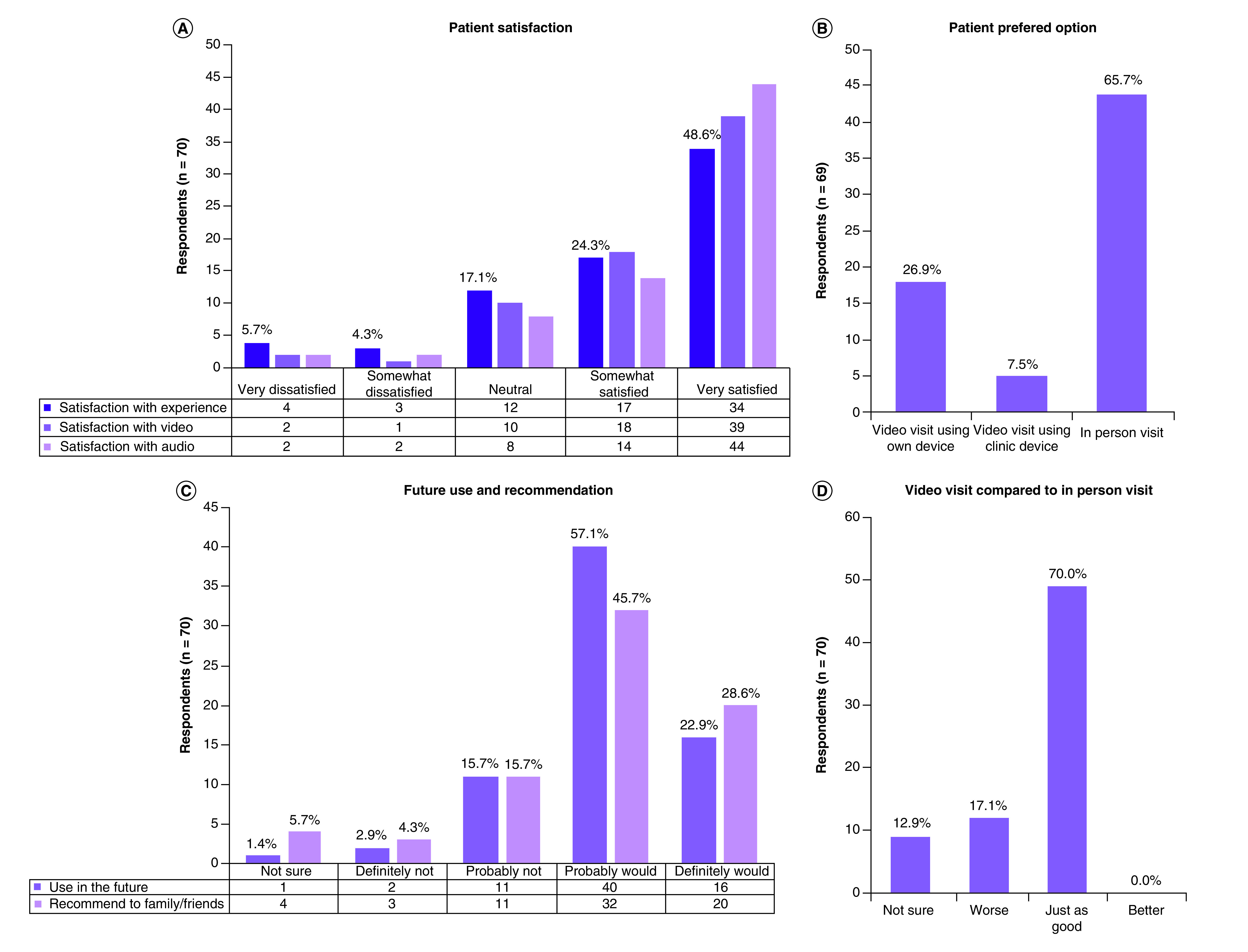
Patient satisfaction, preferred method, patient referral and comparison. **(A)** Results of primary outcome, patient satisfaction with video visits. **(B)** Patient preferred method of conducting visit. **(C)** Patients who would use video visits in the future and recommend to their family/friends. **(D)** Comparing video visits to in-person visits.

A summary of the results for the primary outcome and other survey questions is shown in Figure 3. For the primary outcome, 72.9% of patients (n = 70) reported that they were ‘somewhat’ or ‘very’ satisfied with video visits. Patients were ‘somewhat’ or ‘very’ satisfied with the video and audio of the visit at a higher rate of about 82.7% for both categories. In assessing patient preferences, if given any option for the method of conducting a prechemotherapy evaluation, 65.2% of patients preferred in-person visits, with the remainder choosing a video visit option. 80% of patients also reported that they probably or definitely would use video visits if it were an option in the future and 77.6% reported that they probably or definitely would recommend video visits to family/friends. While most patients (70%) reported that video visits were just as good as in-person visits, none said that they were better.

## Discussion

We conducted a cross-sectional survey to determine patient satisfaction with telemedicine for prechemotherapy evaluation during the COVID-19 pandemic. The early pandemic period provided a natural experiment for the widespread adoption of telemedicine services at our cancer center; we believe that this study captures patients’ attitudes toward a major shift in the way they were receiving their care. The management of cancer patients through a pandemic is undergoing flux based on consensus and committee recommendations; thus providing data from the period of the pandemic can help guide future recommendations [24].

The study’s results show that 73% of cancer patients undergoing prechemotherapy evaluation were satisfied with the video visit experience. 70% of patients believed that video visits were as good as in-person visits, but 65% of patients stated that an in-person visit was their preferred method. No one rated the video visit as better than an in-person visit. This study’s results are not consistent with the prior reported literature which had higher levels of patient satisfaction [9–11,14,17]. The patient preference in this study was mostly for in-person visits; this contradicts the findings of another study that reported a similar measure and found that patients preferred telemedicine visits to in-person visits [12]. There were also not as many ‘very satisfied’ patients as in another study that used a similar video visit platform in a similar population to the one used in this study [15].

A possible reason for this discrepancy may be that this study was conducted in a setting where access to care was not a barrier. Another reason for this study’s results could be that patients undergoing chemotherapy may have been accustomed to seeing their oncologist in person, given that approximately 90% of patients had been coming to the center prior to the declaration of a public health crisis. Lastly, patients may want to see their physicians specifically for prechemotherapy evaluation in person. In-person evaluations have a different flow than video consultations; if certain patients are more accustomed to in-person encounter, then that may be their preferred method for conducting such a visit. Patients diagnosed with cancer may also want an in-person visit because there could be more physical connections, in the form of an exam or providing consolation, that can only be done in person.

Unique aspects of this study include the population that was studied. This study was conducted at a specialty clinic in which patients have regular access to receive their chemotherapy. Patients included in this study also had conducted video visits within the clinic where their providers were primarily located, which may have contributed to the discrepancy between this study’s results and prior work. If patients were presenting to the clinic where they had previously seen their provider in person, they may have wanted to continue seeing their provider in person rather than through a video visit using a clinic device.

The survey was only given to patients who had prechemotherapy evaluation using a video visit, and patients were not included if they only had an initial consultation or other type of visit. Limitations of the study include the response rate of 30%, which is similar to another recently reported study [15]. A possible reason for this low response rate could have been that not all the patients who met the inclusion criteria may have presented to the infusion center during the time the study was conducted. The survey was only administered in English and a translator was not used to conduct it, so language barriers and their effects on the telemedicine experience were not evaluated and may have contributed to a lower response rate, even though most of the clinic population were English speakers. The low response rate could have biased the results of the study toward a population that was either extremely satisfied or extremely dissatisfied with their telemedicine experience during the early phases of the pandemic. The anonymity of the survey also did not allow us to determine whether there were differences in demographic characteristics or number of telemedicine experiences between respondents and non-respondents.

Not all interactions with people were eliminated in this study, because patients would still be presenting for their chemotherapy infusion at the clinic. This study also did not evaluate outcomes for the patients who utilized telemedicine services, nor did it evaluate the appropriateness of telemedicine visits through the COVID-19 pandemic. Not having a physical exam can limit interactions, but a commonly cited reason for patients not preferring a video visit is the lack of one [15]. Future studies should also evaluate the provider experience with the use of telemedicine to see if their satisfaction correlates with the patient experience.

While patients may be more satisfied with telehealth services for other medical care or if they have difficulty in accessing care, this study shows that not all types of visits would benefit from a telemedicine approach. In the comments, patients seemed to be understanding of the public health crisis and the reasons why telemedicine was needed to practice social distancing guidelines. Commonly cited reasons for dissatisfaction from the comments section of the survey included the lack of a physical touch, difficulties with the connection and the social awkwardness of using a video platform. One patient described the encounter as ‘sterile’. Oncology patients undergoing prechemotherapy evaluation may have a preferred method of conducting a consultation based on their prior interactions with their providers and preconceived ideas of how a clinic visit should be conducted.

Changes to reimbursement for telemedicine visits made by the Centers for Medicare and Medicaid Services will make telemedicine a viable option for conducting a consultation [20,21]. Further research is needed to determine whether telehealth through the COVID-19 pandemic has affected patient outcomes and if such visits are appropriate, because it is unknown whether decreasing interactions with providers helps to reduce transmission of viral respiratory infections. Patients may be willing to adapt the way in which they interact with healthcare systems in a public health crisis, but certain populations may still prefer a more traditional in-person visit for certain medical problems, even during a pandemic. The COVID-19 pandemic has caused the rapid adoption of telemedicine services and this may cause a shift in how patients view the traditional in-person consultation. This could impact how future oncology patients choose to interact with their physicians, or become a sort of norm for new oncology patients. Future oncology practices should continue to offer telemedicine services for patient convenience, but this should not completely replace the opportunity for patients to visit with their treating physician in person. Video visits were not the preferred option for our patient population. This could have been due to patients already presenting to the same clinic to receive chemotherapy and having come to the clinic prior to the pandemic.

## Conclusion

In conclusion, our cross-sectional study showed that patients at the Baylor Scott and White Medical Center Cancer Center were satisfied with telemedicine during the initial phases of the COVID-19 pandemic. Patients preferred in-person visits for prechemotherapy evaluation if given a choice for conducting a visit.

- 70 surveys were collected from June to July, 2020;
- 73% of patients reported satisfaction with telemedicine services;
-  65% of patients preferred an in-person visit for prechemotherapy evaluation.
